# The Texas Medical News to Be Enlarged

**Published:** 1916-10

**Authors:** 


### The Texas Medical News to be Enlarged.

The *Texas Medical News* is to celebrate its twenty-fifth anniversary by changing its name and its policy. Hereafter it will be known as the *Medical Insurance and Health Conservation*, and it will be a journal of national rather than of local or sectional character.

For many months the owner and editor of the *Texas Medical News* has been contemplating this change. He has conferred with some of the most prominent men in the East, North and South, and they are all of the opinion that such a journal will serve a long felt need in insurance circles. In fact, many of the medical directors of the large Eastern insurance companies have expressed satisfaction at its advent, and many of the large insurance organizations have given the new journal their approval and sanction.

It is estimated that at least one-half of the physicians practicing in the United States are either medical directors or local examiners for life insurance companies, and as there is no publication devoted exclusively to medical insurance and health conservation topics, it would appear a splendid field for usefulness. Then, too, the wonderful advances which have been made along the lines of health conservation within the last few years have doubtless made the entire profession feel the need of just such a publication as the journal of *Medical Insurance and Health Conservation.*

The editorial staff is composed of men and women from all parts of the United States. Dr. M. M. Smith, the popular and enterprising editor of the former *News,* will retain the active management of the new journal. The TEXAS MEDICAL JOURNAL extends to the new publication the right hand of fellowship and friendship and, like Rip Van Winkle, hopes that it may live long and prosper.

---

The Weekly Bulletin of the Department of Health of New York, in its issue of September 23d, makes the following report, infantile paralysis; total number of cases, 8861; total number of deaths, 2226.

---

Those who are interested in the study of the internal secretions will be glad to know that a new association has been recently formed for the purpose of correlating the work of physicians and other students of this phase of medicine in different parts of the world.

Dr. Henry Harrower, the secretary of the association, will gladly send a full explanation and will be glad to send literature setting forth the plans and purposes of the organization to those who are interested in this special line of study.

